# Using text-mined trait data to test for cooperate-and-radiate co-evolution between ants and plants

**DOI:** 10.1371/journal.pcbi.1007323

**Published:** 2019-10-03

**Authors:** Katrina M. Kaur, Pierre-Jean G. Malé, Erik Spence, Crisanto Gomez, Megan E. Frederickson

**Affiliations:** 1 Department of Ecology and Evolutionary Biology, University of Toronto, Toronto, Ontario, Canada; 2 SciNet Consortium, University of Toronto, Toronto, Ontario, Canada; 3 Departament Ciències Ambientals, Universitat de Girona, Girona, Spain; University of Tennessee, UNITED STATES

## Abstract

Mutualisms may be “key innovations” that spur lineage diversification by augmenting niche breadth, geographic range, or population size, thereby increasing speciation rates or decreasing extinction rates. Whether mutualism accelerates diversification in both interacting lineages is an open question. Research suggests that plants that attract ant mutualists have higher diversification rates than non-ant associated lineages. We ask whether the reciprocal is true: does the interaction between ants and plants also accelerate diversification in ants, i.e. do ants and plants cooperate-and-radiate? We used a novel text-mining approach to determine which ant species associate with plants in defensive or seed dispersal mutualisms. We investigated patterns of lineage diversification across a recent ant phylogeny using BiSSE, BAMM, and HiSSE models. Ants that associate mutualistically with plants had elevated diversification rates compared to non-mutualistic ants in the BiSSE model, with a similar trend in BAMM, suggesting ants and plants cooperate-and-radiate. However, the best-fitting model was a HiSSE model with a hidden state, meaning that diversification models that do not account for unmeasured traits are inappropriate to assess the relationship between mutualism and ant diversification. Against a backdrop of diversification rate heterogeneity, the best-fitting HiSSE model found that mutualism actually decreases diversification: mutualism evolved much more frequently in rapidly diversifying ant lineages, but then subsequently slowed diversification. Thus, it appears that ant lineages first radiated, then cooperated with plants.

## Author summary

Many plants and animals depend on other species for nutrition, protection, or dispersal, a type of ecological interaction known as mutualism. Mutualisms often help organisms thrive in new or harsh environments, thereby increasing their ecological success. We studied whether mutualism also increases evolutionary success by affecting lineage diversification, or the net result of the formation and loss of species over evolutionary time (i.e., speciation minus extinction). We focused on the widespread mutualism between ants and plants, in which ants act as protective ‘bodyguards’ or seed dispersers for plants and gain food or shelter in return. Previous research has found that the evolution of ant-plant mutualisms increased plant diversification. Here, we asked whether the same is true for ant diversification. We used a novel, automated approach to gather trait data from the abstracts of over 89,000 scientific articles about ants, and identified 432 mutualistic ant species and 2,909 non-mutualistic ant species. We then used this trait information to model how mutualism has evolved and influenced diversification across a recent ant phylogeny. Our analysis suggests that instead of causally enhancing diversification, mutualism evolves more often in lineages that are already diversifying quickly and then slows ant diversification.

## Introduction

How have species interactions contributed to the diversification of life on Earth? Ehrlich and Raven [1] famously proposed escape-and-radiate co-evolution as an engine of plant and insect diversification. Here we assess whether “cooperate-and-radiate” co-evolution is also an important diversifying force. Recent studies have linked mutualism evolution to accelerated lineage diversification [2–4], suggesting that mutualism either buffers lineages against extinction, promotes speciation as lineages enter new adaptive zones, or both [5]. Building on previous research showing that partnering with ants enhances plant diversification [3,4], we ask if interacting mutualistically with plants enhances ant diversification.

Mutualism theory generally predicts the opposite; mutualism is expected to hinder diversification [6,7] because the interdependence of partners, conflicts of interest between them, or the invasion of selfish “cheaters” should make mutualistic lineages vulnerable to extinction [8–10]. However, the available phylogenetic evidence strongly suggests that mutualisms often persist over long periods of evolutionary time, and may help lineages flourish [8]. Furthermore, recent studies show that mutualism can expand a lineage’s realized niche [11,12], potentially creating ecological opportunity.

Ant-plant interactions are classic examples of mutualism that have evolved numerous times in both partner lineages [3,4,13]. Ant “bodyguards” visit extrafloral nectaries (EFNs) on plants or nest in specialized plant cavities (domatia) and protect plants against herbivores or other enemies [14]. Ants also disperse seeds that bear lipid-rich elaiosomes [3]. The evolution of elaiosomes [3] and EFNs [4], but perhaps not domatia [13], enhances plant diversification, providing one possible explanation for the rapid radiation of angiosperms famously referred to as an “abominable mystery” by Darwin [15]. Ants can reduce the negative effects of seed predators or herbivores on plant populations, potentially reducing extinction risk [4,16], or they may help plants colonize new sites, promoting speciation [3,16], although it is worth emphasizing that ants move seeds only short distances [17]. Since ant and angiosperm radiations are broadly contemporaneous, having diversified during the Late Cretaceous [18–20], they may have ‘cooperated-and-radiated’; on the ant side, the evolution of ant-plant interactions may have made new niches available in the form of plant-derived food or nest sites, resulting in expanded ranges or decreased extinction risk. However, Nelsen et al. [21] recently found that diet and arboreality did not influence ant diversification; although they did not test for the effect of mutualism with plants *per se*, living and feeding on plants did not have an effect on ant diversification in their genus-level analysis.

To investigate cooperate-and-radiate co-evolution at a finer scale, we automated the compilation of trait data from the primary literature. Inspired by recent studies that have leveraged advances in bioinformatic pipelines [22] and machine reasoning [23] to characterize, for example, protein-protein interaction networks [24], we took an innovative text-mining approach to compile ant-plant interaction data from the abstracts and titles of over 89,000 ant-related publications. We groundtruthed the results by manually checking a subset of abstracts for false positives and evaluated how the number of unique plant-ant species identified by our text-mining algorithm changed as the algorithm sampled more abstracts. We analyzed our trait data in conjuction with a species-level ant phylogeny using Binary State Speciation and Extinction (BiSSE) [25], Bayesian Analysis of Macroevolutionary Mixtures (BAMM) [26], and Hidden State Speciation and Extinction (HiSSE) [27] models to assess the effect of mutualism on ant diversification. Thus, we use a text-mined trait dataset to evaluate whether the evolution of mutualism with plants has spurred ant diversification. In combination with previous research, finding evidence for mutualism-driven diversification would suggest that ants and plants have diversified in tandem through cooperate-and-radiate co-evolution.

## Methods

### Sources of phylogenetic information

We used the Nelsen et al. [21] phylogenetic tree that includes 1,731 ant species, applying the drop.tip function in the R package *ape* [28] to include only tips for which we had trait data. The tree is a compilation of previously published ant trees and provides the most up to date inference of evolutionary relationships among ants. However, since there are 14,416 recognized valid ant species names [29], even a tree with 1,731 ant species is woefully under sampled. The recent phylogenetic comparative methods we used account for incomplete taxon sampling, but these methods are not without error [26]. We did not account for phylogenetic uncertainty, as such analyses are computationally intensive for many phylogenetic comparative methods; our branch lengths and consequent diversification estimates may therefore be affected.

### Sources of trait data

We text-mined 89,495 titles and abstracts from two sources, after removing duplicates prior to analysis: 1) 62,988 abstracts from *FORMIS*: A Master Bibliography of Ant Literature, containing all known ant literature through to 1996 [30] (accessed Oct 2016), and 2) 52,885 ant-related publications available through Springer’s Application Portal Interface (API) (accessed April 2016); Springer publishes numerous journals with substantial ant-related content, including *Oecologia*, *Insectes Sociaux*, *Arthropod-Plant Interactions*, and others. We used Springer’s API to search titles and abstracts for ant species names and plant traits that facilitate ant-plant mutualisms (S1 Table). We downloaded the abstracts of the Springer articles that mentioned the ant species or trait terms on our list. One of us (CG) provided a hand-compiled list of seed-dispersing ants from reading 180 journal articles; we supplemented the text-mining results with data from this hand-compiled dataset.

### Text-mining approach

Because a significant amount of information can be discerned from word combinations alone [31], text-mining can be an effective tool to extract information from the published scientific literature. We took a text-mining approach to compile trait data on ant-plant associations; specifically, to determine which ant lineages consume food bodies, nest in domatia, visit EFNs, and disperse seeds. We created a term-document matrix to hold the trait data using the Pandas and NumPy packages in Python 2.7.12 [32,33]. Our Python script used an *n*-gram approach that allowed us to identify short sequences of words, such as ant binomial nomenclature names or trait terms [31]. Our n-grams were: 1) all 14,416 currently valid ant species names in a global list of ants [29], excluding extinct taxa, and 2) a list of traits related to ant-plant mutualisms (S2 Table). The resulting term-document matrix identifies whether 1) each ant name appeared without a trait or 2) each ant name appeared with a trait, in each publication’s title or abstract. We text-mined trait terms in four broad categories (domatia, extrafloral nectar, food bodies, and seed dispersal) to capture many different types of ant-plant mutualisms. We used the term-document matrix to score each mutualism category as a discrete binary trait for each ant species, and then further combined all the data into a single binary “plant mutualist” category. This approach assumes that ants that nest in domatia, visit EFNs, collect food bodies, or disperse seeds are generally mutualistic, as better data on partner quality are not available at such a broad phylogenetic scale; this assumption is supported by several meta-analyses that have found that ant bodyguards are, on average, beneficial to plants [34–36].

### Text-mining validation

If an ant species name never occurred in the same abstract as a trait term (S2 Table), but appeared in at least one abstract without a trait term, it was scored as an absence (0). If an ant species name co-occurred with a trait term in at least one abstract, it was scored as a presence (1). Both false positives and false negatives are a concern with text-mined trait data, although it is worth noting that other methods of gathering trait data can also be error-prone at such a large scale. Nonetheless, we validated our text-mined trait data in several ways. First, we scrutinized cases in which ant species names co-occurred with trait terms in 5 or fewer abstracts. We read each of these abstracts and manually scored the ant species in question as a true (1) or false (0) association with the trait term. We compared these manual scores to the text-mining results to determine how the number of false positives declined as the number of abstracts containing ant names and trait terms increased. The trait data for the manually checked abstracts were corrected if required for all subsequent analyses. Although we removed most duplicate abstracts from our corpus during initial processing, because not all abstracts were identically formatted, we discovered a few additional duplicates while reading abstracts and adjusted the dataset accordingly. We also calculated species accumulation curves, in which we determined how the number of unique plant-ant and non-plant-ant species increased as a function of the number of abstracts that were text-mined. Finally, we also compared the text-mined data to our hand-compiled data set; to compile the latter, one of us (CG) read 180 articles specifically chosen because they focus on myrmecochory, or seed dispersal by ants, and manually scored which ant species disperse seeds.

### Lineage diversification analyses

We assessed the influence of being a plant-mutualist on ant diversification in several ways. Working in R [37], we used Binary State Speciation and Extinction [25] (BiSSE) models implemented using both the *diversitree* [38] and *hisse* [27] packages and Hidden State Speciation and Extinction (HiSSE) models implemented using the *hisse* [27] package. We also fit Bayesian Analysis of Macroevolutionary Mixtures (BAMM) models using bamm 2.5.0 and *BAMMtools* [39] in R [37].

BiSSE estimates the transition rate between states (q_01_ and q_10_) as well as state-specific extinction and speciation rates (mu_0_ and mu_1_ and lambda_0_ and lambda_1_, respectively). We ran a BiSSE model with a specified root state of 0 (i.e,. non-mutualistic); this is reasonable because the ancestor of all extant ants was probably not a plant mutualist [21]. To calculate state-specific sampling fractions, we first estimated the total number of ants that engage in plant mutualisms by multiplying the proportion of mutualistic ants in our trait dataset (mutualistic ants/total ants in trait dataset = 432/3341, see Results) by the total number of currently valid ant species names (14,416) resulting in an estimated 1,864 ant species that are plant mutualists and 12,552 ant species that are not. We then specified the state-specific sampling fractions as the number of non-mutualistic tips in the phylogeny divided by the estimated total number of non-plant-ants (12,552) and the number of mutualistic tips in the phylogeny divided by the estimated total number of plant-ants (1,864). The results were qualitatively similar when using a global sampling fraction of simply the number of tips in the tree over the total number of currently valid ant species. To test whether mutualist and non-mutualist lineages have different diversification rates, we ran a Markov Chain Monte Carlo (MCMC) BiSSE analysis in *diversitree* [38] with an exponential prior with rate 1/2r, where r is the independent diversification rate of the character. An initial MCMC was run with a tuning parameter of 0.1 for 1,000 generations. The revised tuning was calculated from the width of the middle 90% of the posterior samples from these initial runs. The MCMC analysis was subsequently run for 10,000 generations. We estimated net diversification (speciation–extinction) rates in mutualistic and non-mutualstic ant lineages. We calculated 95% credible intervals of the posterior samples for each parameter from the BiSSE run.

Rabosky and Goldberg [40] showed that low transition rates and rare rate shifts can cause BiSSE (or similar) models to favor state-dependent diversification over a null model. They also highlighted a model inadequacy in which diversification rate heterogeneity is spuriously attributed to neutral traits. Thus, Beaulieu and O’Meara [27] recently developed HiSSE, in part to address some of these issues with earlier SSE models. HiSSE models unobserved, or “hidden,” rate classes with potentially different transitions to or from the trait of interest (here, mutualism with plants) [27]. In other words, this class of models allows there to be rapidly diversifying parts of the tree and slowly diversifying parts of the tree, and these may differ in their trait-dependent speciation, extinction, or transition rates. BiSSE tests for only a single rate category (i.e., it models different transition, speciation, or extinction rates associated with the presence or absence of a measured trait) and is a special case of the HiSSE model, which includes one or more hidden states in addition to the observed states tested in BiSSE.

We ran several HiSSE models that estimated speciation, extinction, and transition rates between states 0 (non-mutualist) and 1 (mutualist) in two rate classes, A and B, for a total of four states: 0A, 1A, 0B, and 1B. Again, we specified the root as state 0 and used the same sampling fractions as in the BiSSE model in *diversitree* [38]. We constrained transitions among states differently in each model by supplying a different transition matrix: we ran two BiSSE models in *hisse* [27] (a null model with no hidden states and a trait-dependent model with no hidden states), a CID-2 HiSSE model (trait-independent diversification with two hidden states), a CID-4 HiSSE model (trait-independent diversification with four hidden states), a HiSSE model with one hidden state, and a full, two hidden state HiSSE model. The latter two models allow speciation, extinction, and transition rates to vary with both observed traits and hidden states. We ran these models using the combined plant mutualist category, as well as separated into defense and seed dispersal mutualisms.

Finally, we also ran BAMM analyses three times on the same tree. The BAMM was run for 10 million MCMC generations, sampling the parameters after every 100,000 generations. To indicate how much clade information was missing to account for incomplete taxon sampling, we included a proportion for each ant genus. It was calculated as the number of species in a particular genus in the tree over the total number of species in the genus (S1 Text). Rate priors were calculated for each tree using *BAMMtools* [39] and convergence of the BAMM runs was also tested using the R [37] package *coda* [41]. To assess if diversification rates differed in mutualistic and non-mutualistic ant lineages, we used the subtreeBAMM and getcladerate functions in *BAMMtools* [39] to assess whether areas of the tree in the mutualist state had higher diversification rates than areas of the tree in the non-mutualist state. We used the BAMM output to assess the diversification rate of mutualistic and non-mutualistic ant lineages. We also ran a two-tailed STRAPP (Structured Rate Permutations on Phylogenies) analysis on the BAMM output for 10,000 replicates using the traitDependentBAMM function in *BAMMtools* [39].

## Results

**Fig 1:**
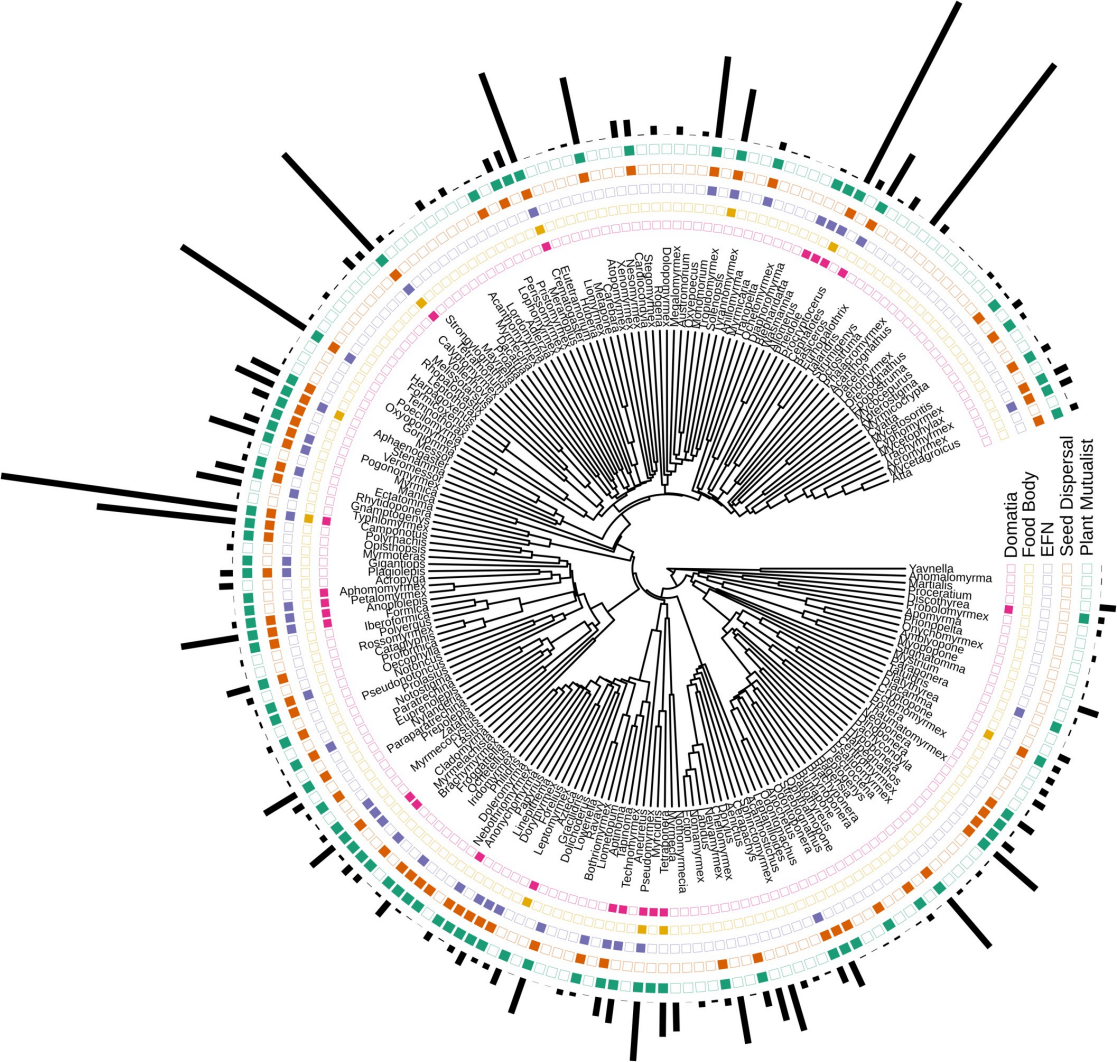
Visualization of trait data, showing which genera (N = 199) contain species that nest in domatia (pink), consume food bodies (yellow), visit EFNs (purple), disperse seeds (orange), or engage in any plant mutualism (combination of all traits) (green). Black bars show the number of species in each genus.

**Fig 2:**
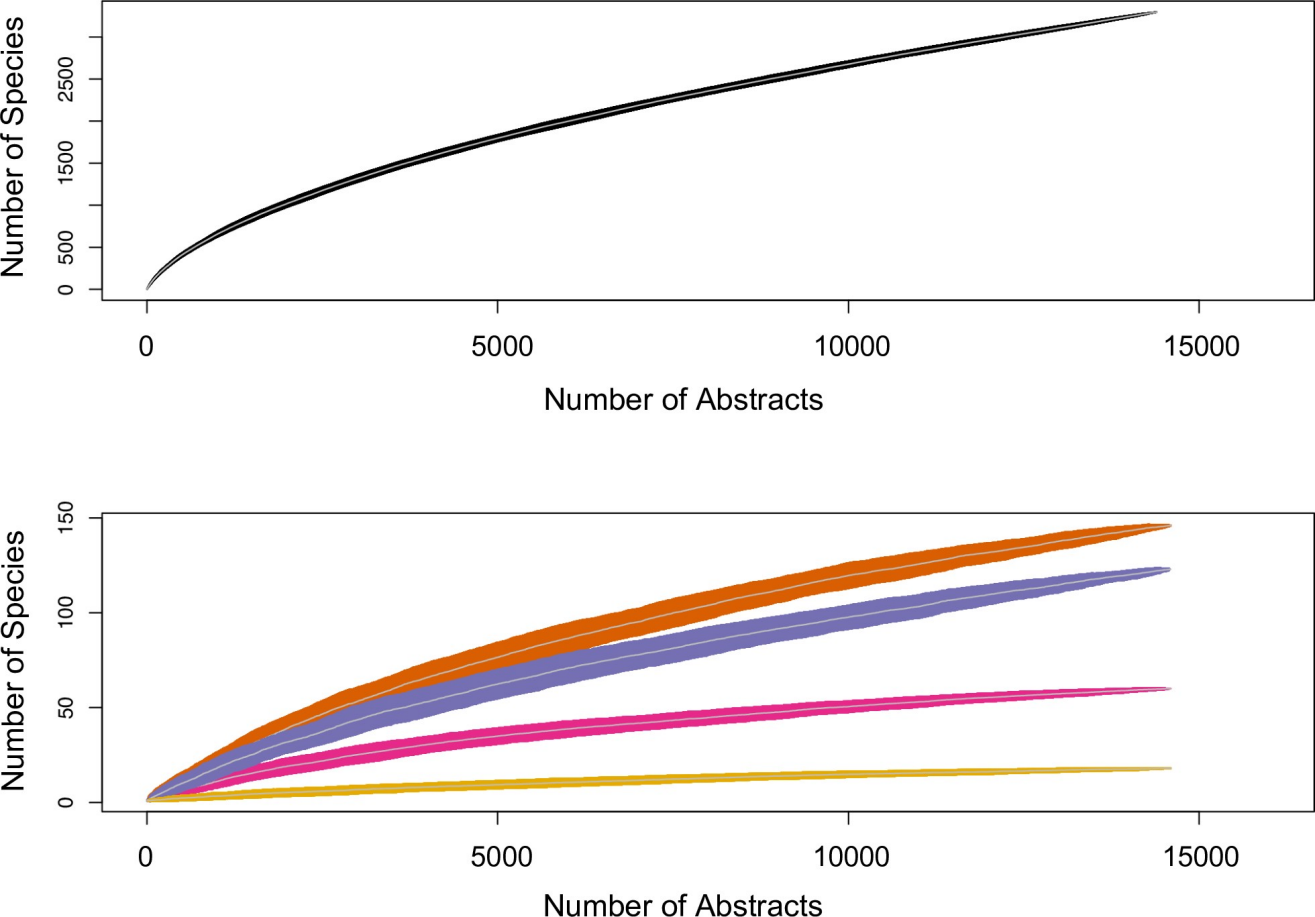
Species accumulation curves showing how the number of unique ant species increased as more abstracts were sampled for both ants that do not interact mutualistically with plants (top panel) and ants that disperse seeds (orange), visit EFNs (purple), nest in domatia (pink), and consume food bodies (yellow) (bottom panel). Nearly 15,000 abstracts contained ant names. Grey lines are means and black or colored regions are standard deviations calculated from sub-sampling abstracts 100 times at each x-axis value.

Text-mining generated a wealth of trait data (Fig 1, S1 Fig). This method outputted trait data for 3341 ant species in 265 ant genera, representing 23% and 73%, respectively, of all currently recognized ant species and genera, globally. The text-mining extracted these data from approximately 15,000 abstracts, with the number of unique species increasing as the number of abstracts sampled increased (Fig 2). The species accumulation curves suggest that our lists of ant species that nest in domatia or consume plant food bodies are relatively complete as we are unlikely to identify many more ant species for these traits, even with the addition of more abstracts. However, text-mining an even a larger sample of abstracts from the primary literature would likely identify many more ant species that disperse seeds or visit EFNs, as well as many more non-mutualistic ant species (Fig 2).

We paired this approach with a more traditional method of manually assembling a list of seed-dispersing ants by reading the primary literature. Without knowing what abstracts were in the corpus we text-mined, one of us (CG) identified 180 articles on myrmecochory and hand-compiled a list of seed-dispersing ants. This allowed us to directly compare the effectiveness of our method of text-mining abstracts and an independent manual compilation of trait data from full texts. By comparing a hand-curated data set to a text-mined data set we can assess the differences in these two approaches to collecting trait information. Our automated approach functioned relatively well in comparison; 85 seed-dispersing ant species in 28 genera occurred in both our text-mining results and this hand-compiled dataset. In total, the text-mining identified 129 ant species in 39 genera as seed dispersers, compared to 268 ant species in 60 genera in the hand-compiled dataset, meaning that 44 ant species were in the text-mining results only, and 183 species were in the hand-compiled dataset only. Of the 183 seed-dispersing ant species in the hand-compiled dataset but not in our text-mining output, 149 species were described in 92 papers that were not included among our 89,000 abstracts, suggesting that the largest improvements to our text-mined trait dataset would come from having access to more abstracts, rather than from a better text-mining algorithm. The text-mining method was not immune to error, but the number of false positives declined rapidly as the number of abstracts in which ant names and trait terms co-occurred increased. We found no false positives when an ant species name co-occurred with one or more trait terms in 4 or 5 abstracts (S2 Fig). We used the entire trait dataset in our lineage diversification analyses, but only after removing the false positives we found by manually checking ant names that co-occurred with trait terms in 3 or fewer abstracts.

Seed-dispersing and EFN-visiting ants were most commonly identified by text-mining, followed by domatia-nesting ants; relatively few ant species that consume food bodies were found by text-mining. Specifically, we identified 309 ant species in 77 genera that disperse seeds (including taxa in the hand-compiled dataset) and 3030 ant species in 261 ant genera that do not; 122 ant species in 37 genera that visit EFNs and 3182 ant species in 261 ant genera that do not; 58 ant species in 22 genera that nest in domatia and 3246 ant species in 262 genera that do not; 16 ant species in 8 genera that consume plant food bodies (other than elaiosomes) and 3288 species in 265 genera that do not. In all, we identified 432 ant species in 84 genera that are plant mutualists, and 2909 ant species in 256 genera that are not.

After pruning the phylogeny to match the trait data, the tree was comprised of 795 species in 199 genera (Fig 1, S1 Fig). Unlike other studies of trait-dependent diversification, we did not include species for which we had no trait information (i.e., ant species that did not appear in our text-mined abstracts). Thus, the 795 species used in the BiSSE, HiSSE, and BAMM analyses were species for which the text-mining determined the ant species is likely a plant mutualist (195 species), as well as species for which the text-mining determined the ant species is likely not a plant mutualist (600 species); the latter ant species names appeared in at least one of our ~89,000 abstracts but were never found together with any plant mutualist terms (S2 Table).

**Fig 3:**
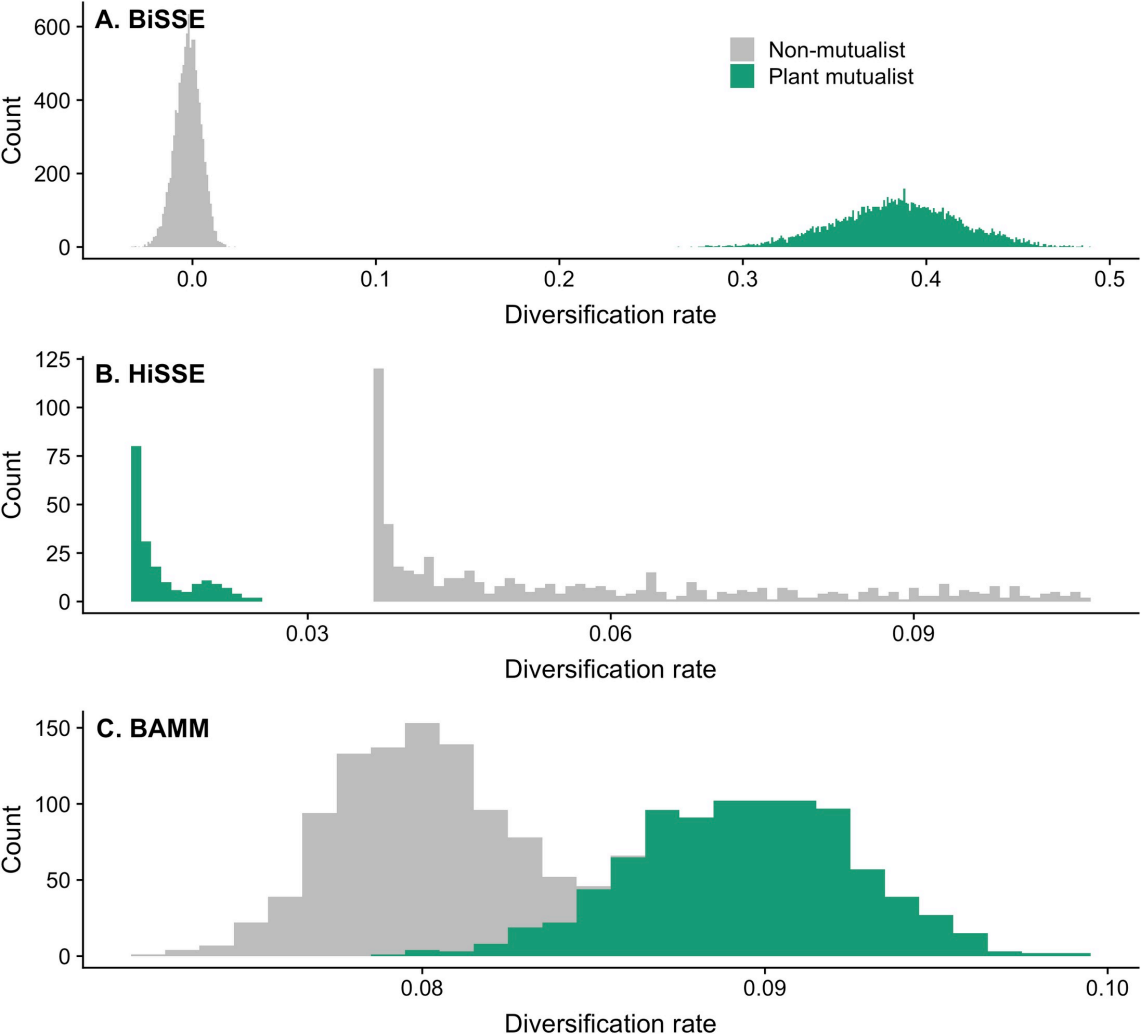
Diversification rates from (A) BiSSE in *diversitree*, (B) the best-fitting full HiSSE model in *hisse*, and (C) BAMM analyses for ant lineages that are plant mutualists (green) and ant lineages that are not plant mutualists (grey). HiSSE output represents average rates for tip states for all 795 tips on the phylogeny. BAMM output represents one BAMM run and diversification rates on areas of the tree with and without the trait.

The BiSSE model in *diversitree* [38] found that mutualistic ant lineages diversify faster than non-mutualistic ant lineages. There was no overlap between plant mutualists and non-mutualists in their 95% credible intervals for speciation, diversification, and transition rates, meaning these differences are statistically significant, while 95% credible intervals for extinction rates overlapped between traits (S3 Table). BiSSE reported a non-significantly lower extinction rate but a significantly higher speciation rate in mutualistic than non-mutualistic ants, resulting in much faster diversification in mutualistic ants (Fig 3).

**Fig 4:**
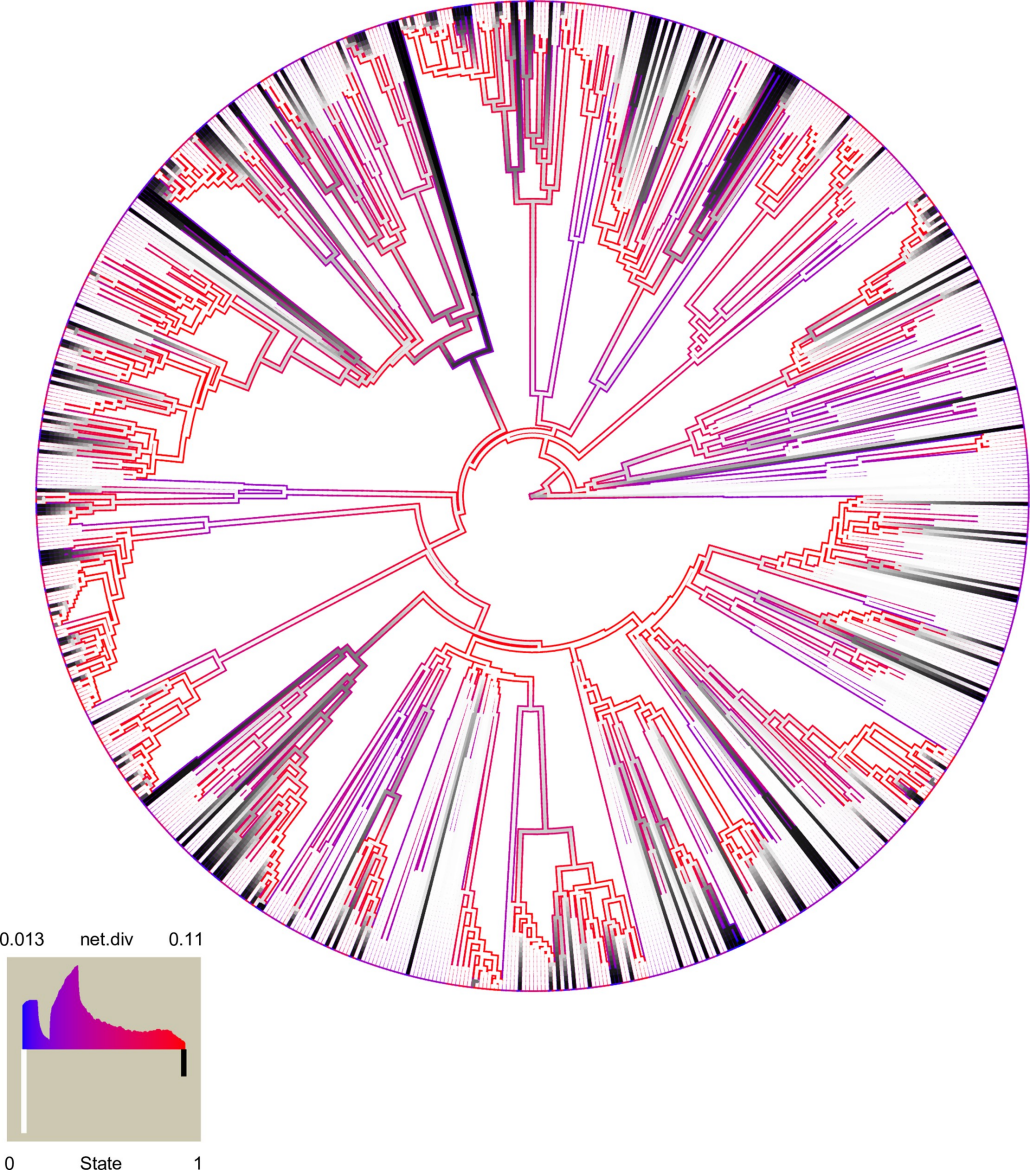
Net diversification rate from full HiSSE model with four rate classes (0A, 1A, 0B, 1B) mapped as a continuous trait on the pruned phylogeny. More slowly diversifying lineages are blue and more rapidly diversifying lineages are red, as in inset Fig. Plant mutualist state is coloured white (state 0 or non-mutualist) to black (state 1 or mutualistic with plants).

The HiSSE analysis found strong support for ‘hidden’ rate classes in the ant tree, with some parts of the tree diversifying much faster (rate class B) than others (rate class A) (Table 1, Fig 4). The best-fitting of all the models we implemented in the *hisse* [27] package was the full HiSSE model with a total of four diversification rates (in states 0A, 1A, 0B, and 1B). This model had a considerably lower AIC score than alternative models; model results including speciation, extinction, transition rates, and AIC scores are presented in Table 1 for the combined plant mutualist category. In particular, the four-rate model fit considerably better than the BiSSE models implemented in *hisse* [27]. We found qualitatively similar results when analyzing ant ‘bodyguards’ (i.e., ants associated with EFNs, food bodies, or domatia) separately from seed-dispersing ants (S4 and S5 Tables). In the strongly supported full HiSSE model, mutualism is substantially more likely to evolve in the rapidly diversifying rate class (B) than in the slowly diversifying rate class (A) (i.e., the transition rate from 0B to 1B is many orders of magnitude higher than from 0A to 1A), but once mutualism evolves, diversification actually slows down (Table 1, Fig 4). When rates are averaged over observed states only (0 and 1), there is higher diversification in the non-mutualistic states (Table 2, Fig 3).

**Table 1:** Parameter estimate summary from HiSSE analyses for all models for the plant mutualist category. For the CID-4 model, not all transition rate categories are shown in the table because all 32 transition rates for this model are equal. The following transition rates are removed from all HiSSE models: q1B0A, q0B1A, q1A0B, and q0A1B, as they are dual transitions between both the observed trait and the hidden trait.

	Rate Class	lambda	mu	div		q10	q01	q0A1A	q0A0B	q1A0A	q1A1B	q0B0A	q0B1B	q1B1A	q1B0B
**BiSSE no hidden states** AIC 7927.076	0	0.6335	0.5984	0.0351		0.173	0.0279	not applicable							
	1	0.0453	9.34E-11	0.0453											
**BiSSE null** (lamba0 = lamba1, mu0 = mu1) AIC 7969.408	0	0.448	0.409	0.0390		0.0762	0.00861								
	1	0.448	0.409	0.0390											
**CID-2 (Null-two) HiSSE** (lamba0A = lambda1A, mu0A = mu1A, lambda0B = lambda1B, mu0B = mu1B) AIC 7879.975	0A	0.0449	1.03E-10	0.0449		not applicable		0.00762							
	1A	0.0449	1.03E-10	0.0449											
	0B	0.9035	0.8497	0.0538											
	1B	0.9035	0.8497	0.0538											
**CID-4 (Null-four) HiSSE** (lamba0A = lambda1A, mu0A = mu1A, lambda0B = lambda1B, mu0B = mu1B, lamba0C = lambda1C, mu0C = mu1C, lambda0D = lambda1D, mu0D = mu1D) AIC 7759.882	0A	0.04968	0.00294	0.0467				0.0122 (this model has 32 transitions, due to the added C and D rate classes)							
	1A	0.0497	0.00294	0.0467											
	0B	0.0460	9.48E-11	0.0460											
	1B	0.0460	9.48E-11	0.0460											
	0C	0.5485	0.488	0.0605											
	1C	0.5485	0.488	0.0605											
	0D	1.1385	1.09	0.0485											
	1D	1.1385	1.09	0.0485											
**HiSSE 1 hidden state** AIC 7638.354	0A	0.1937	0.2165	-0.0228				0.0178	0	0.511	0.0237	0	0	0.468	0
	1A	0.365	8.66E-10	0.365											
	0B	0	0	0											
	1B	0.817	0.448	0.369											
**Full HiSSE** AIC 7572.166	0A	0.07913	0.04658	0.0326				2.06E-09	0.00777	0.00343	2.06E-09	0.0554	0.0377	0.00845	0.0579
	1A	0.02984	0.005088	0.0248											
	0B	1.069	0.960	0.109											
	1B	0.2134	0.2008	0.013											

**Table 2:** Parameter estimate summary from MCMC for BiSSE analysis in *diversitree*, the best-fitting full HiSSE model, and post-burn-in MCMC result summary for BAMM analysis. The mean and (standard deviation) of parameters are reported.

	BiSSE in *diversitree*	HiSSE full model	BAMM
**Speciation**_**0**_	0.130 (0.020)	0.404 (0.280)	0.125 (0.0073)
**Speciation**_**1**_	0.457 (0.045)	0.169 (0.0493)	0.153 (0.010)
**Extinction**_**0**_	0.133 (0.022)	0.346 (0.258)	0.0455 (0.0082)
**Extinction**_**1**_	0.0735 (0.059)	0.153 (0.0525)	0.0639 (0.0011)
**Q**_**01**_	0.00900 (0.0017)	-	-
**Q**_**10**_	0.406 (0.039)	-	-
**Diversification**_**0**_	-0.00300 (0.0069)	0.0575 (0.0215)	0.0795 (0.0022)
**Diversification**_**1**_	0.383 (0.033)	0.0155 (0.00328)	0.0891 (0.0032)
**Overall Rate**_**1-0**_	0.386 (0.037)	-0.0420 (-0.0182)	0.0096 (0.0023)

The BAMM analysis reported higher speciation and higher extinction rates when ants evolved mutualisms with plants; however, overall diversification rates were higher in mutualistic ants, despite elevated extinction rates, because of the even greater difference in speciation rates between mutualistic and non-mutualistic ants (Table 2). However, the BAMM STRAPP test was non-significant (*p* = 0.1251) and the diversification rate estimates overlap (Table 2, Fig 3). BAMM speciation rates also indicate more recently evolved taxa tend to have higher diversification rates (S3 Fig).

## Discussion

Our results build on previous research [22,23] showing how automated methods can reliably and efficiently assemble large trait databases for answering macroevolutionary questions. Text-mining generated trait data for almost twice the number of ant species as in the most comprehensive phylogeny available, and we had overlapping trait data and phylogenetic information for 795 ant species. We used these data to test whether plant-ant lineages have elevated diversification rates, a hypothesis that was supported by the BiSSE model implemented in *diversitree*. However, the best-fitting HiSSE model found strong evidence for a “hidden” state influencing diversification, meaning that the effect of mutualism on diversification must be assessed in light of underlying rate heterogeneity, something not possible with BiSSE or BAMM. The most strongly supported model was the four-rate, full HiSSE model in which both a hidden state and mutualism have influenced ant diversification. Specifically, this model found that mutualism tends to evolve in rapidly diversifying clades and then slows diversification. Thus, the HiSSE model found a more complex relationship between mutualism evolution and lineage diversification than previous work has suggested, but nonetheless indicated that they are interdependent.

Text-mining was a successful and efficient method for assembling a large trait database, and was primarily limited by the availability of abstracts. The species accumulation curves (Fig 2) suggest that with an even larger corpus, we could acquire trait data for many more ant taxa, and especially that we would identify many more EFN-visiting and seed-dispersing ant species. Our text-mining extracted similar trait information as what is normally assembled manually, and more laboriously, from the primary literature; an automated approach could also vastly improve datasets for meta-analyses, ecological network analysis, etc. Although our text-mining algorithm returned a small number of false positives because we took any co-occurrence of a trait term and an ant species name in an abstract as evidence of an association, the number of false positives declined rapidly as trait terms and ant names were found together in more abstracts (S2 Fig), again suggesting that a larger corpus would help to reduce noise in the text-mining output. We also text-mined only abstracts; assuming abstracts of papers on ant-plant mutualisms are less likely than main texts to mention non-mutualistic ants in passing, our method should be more conservative regarding false positives, but of course full-text articles contain more trait information. To improve on our text-mining method, considering the proximity between words or the frequency of words, or a more restrictive rule for how often an ant name and trait term need to co-occur, could help to further reduce the frequency of false positives. However, a more restrictive rule may not be necessary with a sufficiently large corpus, as our comparison between the text-mined and hand-compiled data sets shows that most of the seed-dispersing ants missed by the text-mining were described in papers not in our corpus.

Automated downloading of large numbers of abstracts proved difficult as most are behind paywalls, and even with institutional subscriptions, it is challenging to download publications en masse [42]. We chose to text-mine only abstracts because full-text articles are even less easily accessible. Thus, more open access publications could increase the efficacy and benefits of automated data collection methods such as text- or data-mining. Nonetheless, using text-mining to extract data from published papers could be applied to a variety of research questions, given that data is readily available in word combinations. For example, the text-mining algorithm could easily be adopted to also mine plant species names in order to construct large ant-plant networks, permitting exploration of both network structure and co-phylogenetic patterns.

We report both speciation and extinction values from the SSE models (Tables 1 and 2) but focus our discussion on diversification rate estimates. Overall, the BiSSE and BAMM models found that the evolution of ant-plant mutualisms was associated with higher ant lineage diversification; we found a positive effect of mutualism evolution on ant diversification in both analyses, although the effect was non-significant (per STRAPP) in the BAMM analysis (Table 2, Fig 3). Previous research has similarly found that partnering with ants for defense or seed dispersal accelerates plant diversification [3,4]; combined with our BiSSE results, this could suggest that ant and plant lineages are either responding to the same external factor(s) affecting diversification (e.g., biogeography, see below), or that ants and plants cooperate-and-radiate. For example, by engaging in mutualism, ants might increase the size of their geographic range or realized niche, if plant rewards allow ants to live in previously unsuitable habitats. Ecological success might then confer evolutionary success, through faster allopatric speciation or increased population sizes reducing rates of extinction. Similar arguments have been made to explain how the evolution of EFNs and elaiosomes have accelerated plant diversification [3,4].

However, BiSSE often rejects the null model in favor a trait-dependent diversification model whenever diversification rates are highly heterogeneous across the phylogeny, even if diversification is not trait-dependent [27,40]. For this reason, in their recent analysis, Nelsen et al. [21] used HiSSE to evaluate the relationship between nesting or foraging arboreally and ant diversification. They found evidence for heterogeneous diversification rates across the ant phylogeny, but it was not associated with their trait data in any way (i.e., the best-fitting model in Nelsen et al. was CID-4, a character-independent diversification model with four different rate classes). In contrast, with our larger, species-level, text-mined trait dataset, we rejected trait-independent models of diversification in favor of a HiSSE model in which mutualism evolves more frequently in rapidly diversifying lineages, but then subsequently slows lineage diversification. This model suggests mutualism and diversification are not independent, but the relationship is more complex than in BiSSE or BAMM models because of underlying rate heterogeneity. Given that the best-fitting model is the HiSSE model with a hidden state, the positive effect of mutualism on diversification in the BiSSE model from *diversitree* is likely misleading.

The HiSSE model suggests that some ‘hidden’ state may influence both the evolution of ant-plant mutualisms and ant diversification, resulting in the positive association between mutualism and diversification rate in the *diversitree* [38] BiSSE model and, to a lesser and non-signifcant extent, in the BAMM analysis. This state could be biogeographic, for example if ant-plant mutualisms are more likely to evolve in the tropics and ant lineages also diversify more rapidly there, or it could be any number of morphological, behavioral, or life history traits that accelerate diversification and predispose rapidly diversifying ant lineages to mutualism; in reality, probably all of these factors have at least some effect on diversification [27,43] and potentially on mutualism evolution. There is currently substantial interest, but also debate, about ‘drivers’ of diversification [27,40,43], and our results suggest that mutualism’s influence on diversification may need to be considered against a backdrop of rate heterogeneity driven by other, as yet unmeasured, factors. Furthermore, the evolution of mutualism itself may also be contingent on such ‘hidden’ states, but why some lineages evolve mutualism, while others do not, is a question that has received comparatively little attention in the literature [44]. The HiSSE results were not qualitatively different when we modelled ant ‘bodyguards’ separately from seed-dispersing ants (S4 and S5 Tables). This may be because these two types of ants confer very different benefits to plants (defense versus dispersal, respectively) but nonetheless receive similar rewards—mainly food.

However, we hesitate to over-interpret these model results for several reasons. First, in their simulations, Beaulieu and O’Meara [27] found that HiSSE has difficulties adequately estimating transition rates, so perhaps gains and losses of mutualism are more similar across rate classes than the best-fitting HiSSE model indicates. Our transition rate estimates may also be affected by incomplete taxon sampling, given that the phylogeny we used had only 795 of the over 14,000 currently recognized ant species. Relatedly, in both BiSSE and HiSSE models, although the trait evolves independently multiple times, mutualism is very frequently lost (Tables 1 and 2, Fig 4). While this could be taken as evidence that mutualism often breaks down, our trait dataset may include a high number of false negatives that the SSE models reconstruct as secondary losses. If an ant name and trait term co-occur in enough abstracts, there is little doubt that the ant is a plant mutualist, but it is more challenging to be sure that ants named in abstracts that do not also contain trait terms are truly non-mutualistic. Note that this problem persists even though our text-mining approach allows us to be more confident in our trait absences than studies that equate the absence of trait data with the absence of the trait in a taxon. Finally, ant-plant interactions are often diffuse, generalized mutualisms [21] that appear to be easily gained and lost over evolutionary time [13] (Fig 4, see also the BiSSE ancestral state reconstruction in S4 Fig), while SSE models have generally been developed and tested with less evolutionarily labile traits in mind [45].

In summary, our findings suggest that the evolution of ant-plant mutualisms and ant diversification are interdependent, but the relationship may be complex. In combination with previous work on angiosperm diversification, our BiSSE model found support for “cooperate-and-radiate” co-evolution between ants and plants. In contrast, the best-fitting HiSSE model is more consistent with a “radiate-then-cooperate” scenario, with mutualism evolving frequently in rapidly diversifying ant lineages, but then subsequently slowing diversification. But we suspect ours will not be the last word on this subject. We only hope that the next study to assess reciprocal diversification between ants and plants uses text-mining to gather even larger trait datasets that can be modelled on the bigger, better, and more complete ant and plant phylogenies that are surely forthcoming.

## Supporting information

S1 Text BAMM sampling fraction per genus calculated as the number of species in a particular genus in the tree over the total number of species in the genus. (TXT)

S1 Fig Visualization of trait data, showing which species (N = 795) nest in domatia (pink), visit consume food bodies (yellow), EFNs (purple), disperse seeds (orange), or engage in any plant mutualism (combination of all traits) (green).
(PDF)

S2 Fig When an ant species name appeared with a trait term in five or fewer abstracts, abstracts were manually scored to check for false positives. No false positives were detected when ant species name and trait terms co-occurred in at least 4 abstracts.
(TIF)

S3 Fig Phylogeny outputted from plot.bammdata showing speciation rates. (PDF)

S4 Fig BiSSE ancestral state reconstruction, from the asr.marginal function in the R *diversitree* package. Green represents the plant mutualist state and grey represents the non-mutualist state.
(PDF)

S1 Table Trait terms used in conjunction with all currently valid ant species names to extract abstracts from Springer’s API. (DOCX)

S2 Table Trait terms describing ant-plant mutualisms used in the text mining. (DOCX)

S3 Table For the BiSSE model, 95% credible intervals for speciation, extinction, and transition rates of lineages that do not (state 0) or do (state 1) associate mutualistically with plants. (DOCX)

S4 Table Parameter estimate summary from HiSSE analyses for all models for the defense category (which includes trait information for domatia, EFN, and food bodies). For the CID-4 model, not all transition rate categories are shown in the table because all 32 transition rates for this model are equal. The following transition rates are removed from all HiSSE models: q1B0A, q0B1A, q1A0B, and q0A1B, as they are dual transitions between both the observed trait and the hidden trait.
(DOCX)

S5 Table Parameter estimate summary from HiSSE analyses for all models for the seed dispersal category. For the CID-4 model, not all transition rate categories are shown in the table because all 32 transition rates for this model are equal. The following transition rates are removed from all HiSSE models: q1B0A, q0B1A, q1A0B, and q0A1B, as they are dual transitions between both the observed trait and the hidden trait.
(DOCX)
