# Supplementary figures and images for: Using text-mined trait data to test for cooperate-and-radiate co-evolution between ants and plants
Source: PLoS Comput Biol. 2019 Oct 3;15(10):e1007323. doi: 10.1371/journal.pcbi.1007323 (PMC6776258; doi:10.1371/journal.pcbi.1007323)

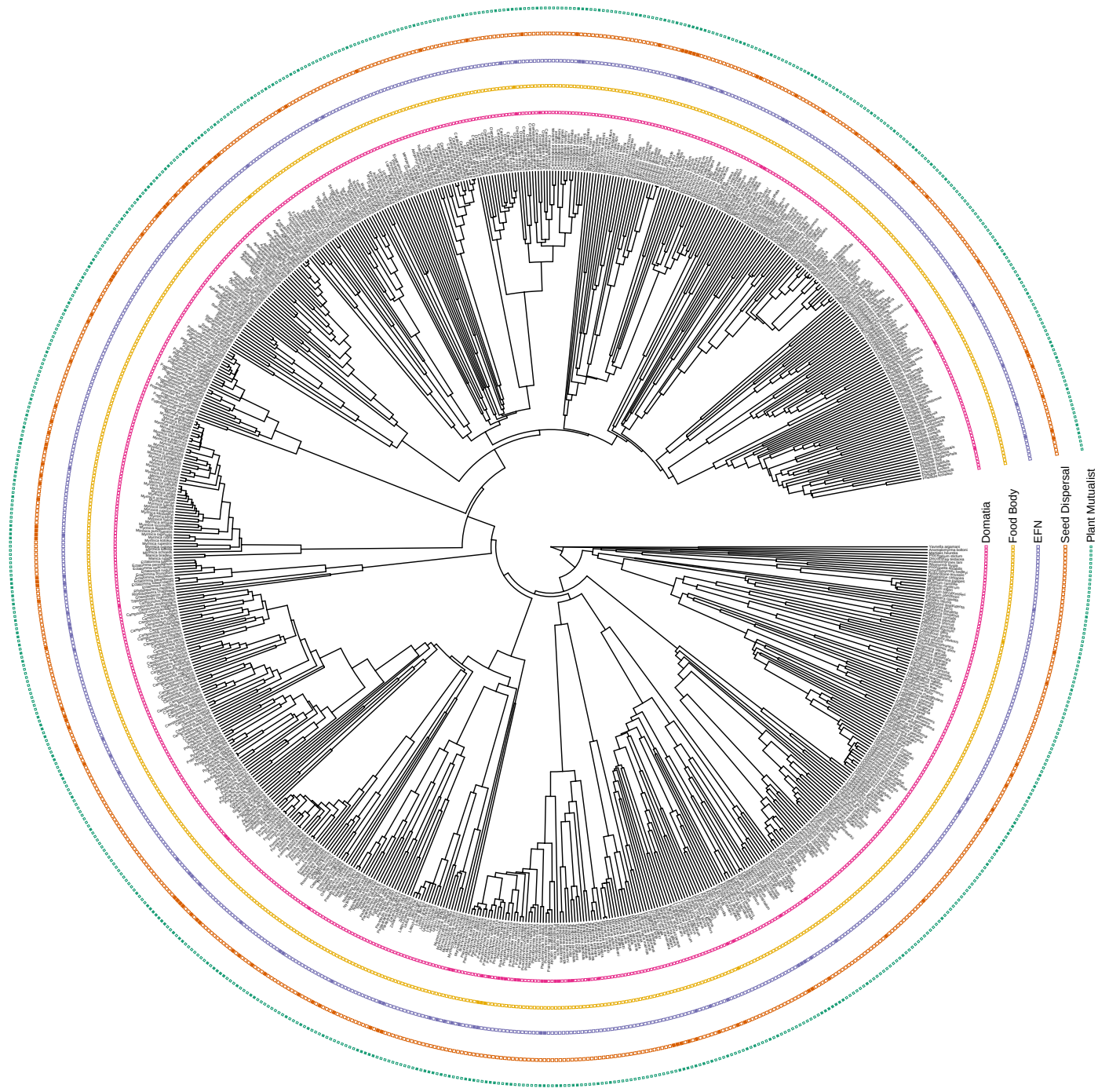

Supplement: S1 Fig — Visualization of trait data, showing which species (N = 795) nest in domatia (pink), visit consume food bodies (yellow), EFNs (purple), disperse seeds (orange), or engage in any plant mutualism (combination of all traits) (green). (PDF) [file pcbi.1007323.s002.pdf]

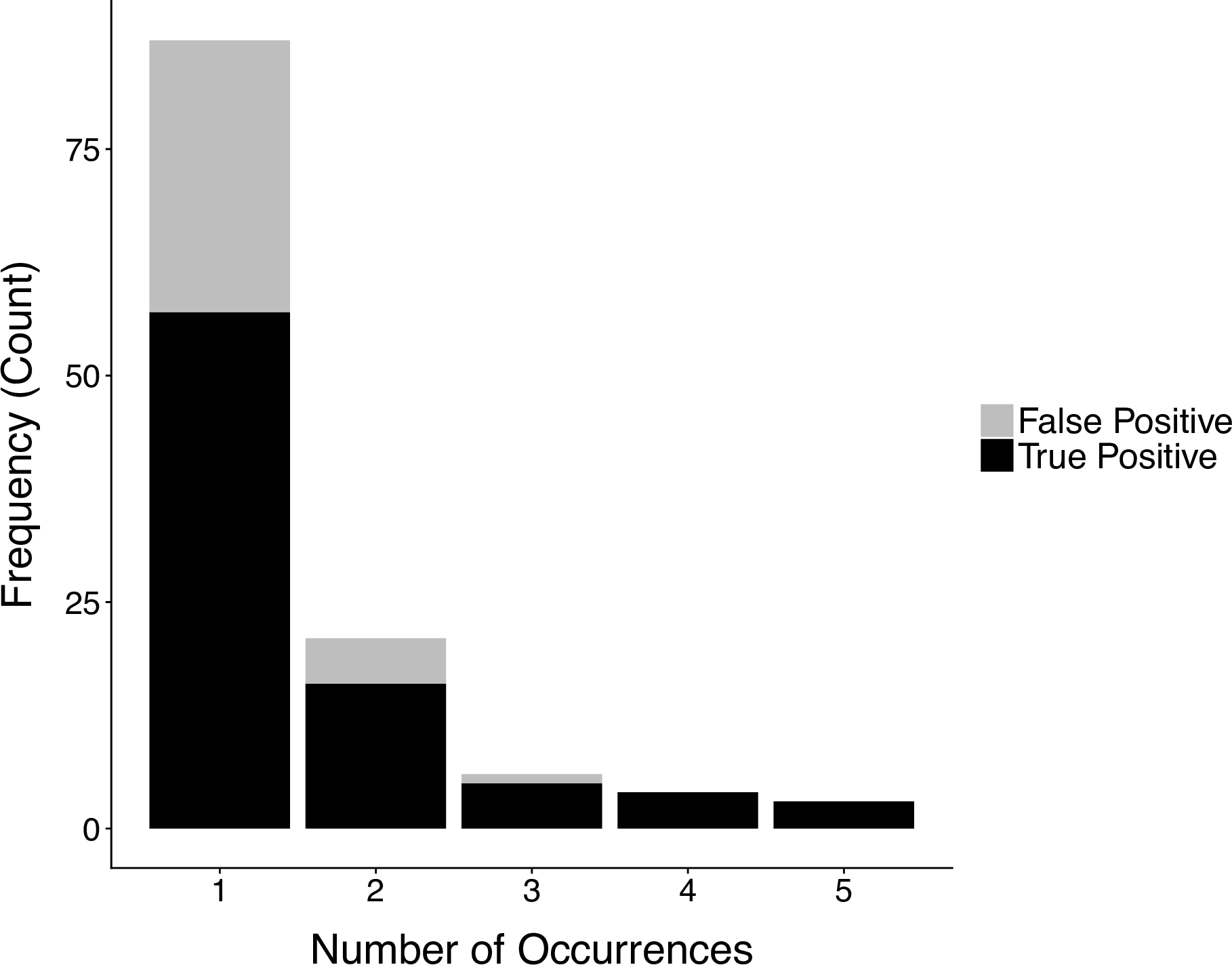

Supplement: S2 Fig — No false positives were detected when ant species name and trait terms co-occurred in at least 4 abstracts. (TIF) [file pcbi.1007323.s003.tif]

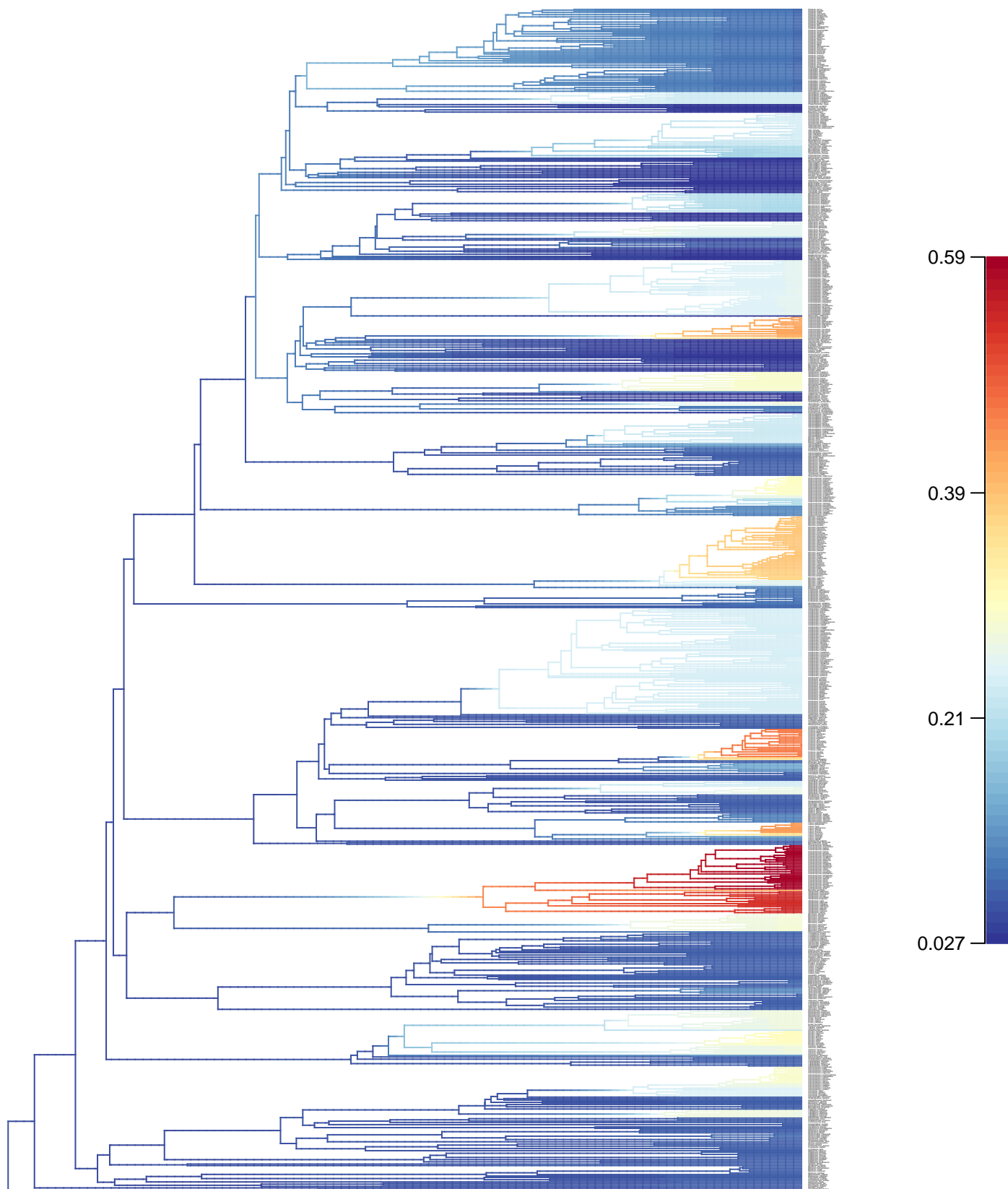

Supplement: S3 Fig — (PDF) [file pcbi.1007323.s004.pdf]

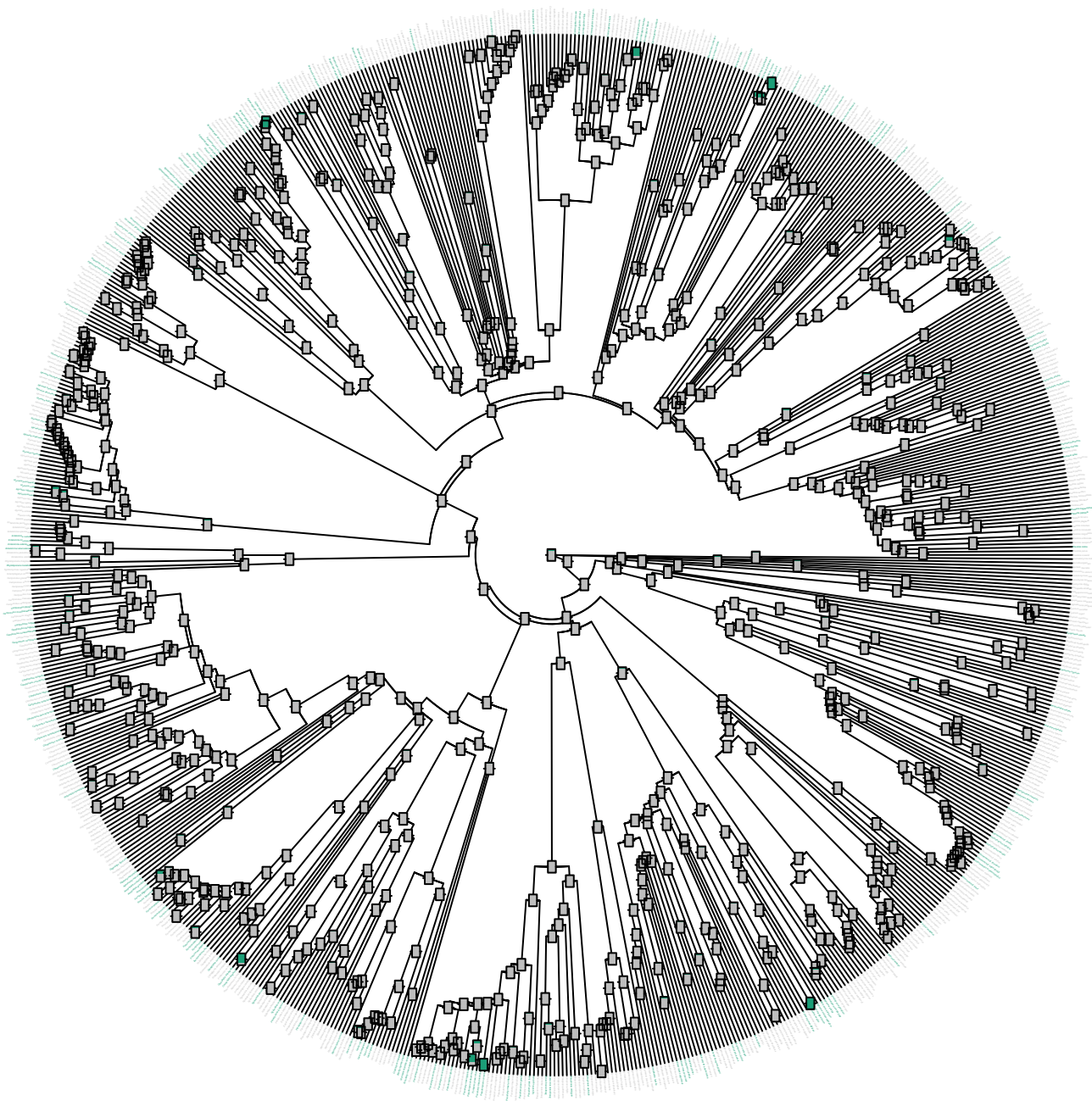

Supplement: S4 Fig — Green represents the plant mutualist state and grey represents the non-mutualist state. (PDF) [file pcbi.1007323.s005.pdf]
