# Supplementary material for: Using text-mined trait data to test for cooperate-and-radiate co-evolution between ants and plants
Source: PLoS Comput Biol. 2019 Oct 3;15(10):e1007323. doi: 10.1371/journal.pcbi.1007323 (PMC6776258; doi:10.1371/journal.pcbi.1007323)
Supplement: S1 Table — (DOCX) [file pcbi.1007323.s006.docx]

*S1 Table*. Trait terms used in conjunction with all currently valid ant species names to extract abstracts from Springer’s API.

| **Broad Category** | **Trait Term** |
| --- | --- |
| **Domatia** | domati* |
|  | leaf pouch* |
|  | myrmecodomati* |
|  | myrmecophyte* |
|  | root tuber* |
|  | trichili* |
| **Extrafloral Nectar (EFN)** | efn* |
|  | extrafloral* |
|  | foliar necta* |
| **Food Bodies** | beltian bod* |
|  | food bod* |
|  | mullerian bod* |
|  | pearl bod* |
| **Seed Dispersal** | aril* |
|  | elaiosome* |
|  | myrmecochor* |
