# Supplementary material for: Using text-mined trait data to test for cooperate-and-radiate co-evolution between ants and plants
Source: PLoS Comput Biol. 2019 Oct 3;15(10):e1007323. doi: 10.1371/journal.pcbi.1007323 (PMC6776258; doi:10.1371/journal.pcbi.1007323)
Supplement: S2 Table — (DOCX) [file pcbi.1007323.s007.docx]

*S2 Table.* Trait terms describing ant-plant mutualisms used in the text mining.

| **Broad Category** | **Trait Term** |
| --- | --- |
| **Domatia** | domatia |
|  | domatium |
|  | leaf pouch |
|  | leaf pouches |
|  | myrmecodomatia |
|  | myrmecodomatium |
|  | myrmecophyte |
|  | myrmecophytes |
|  | root tuber |
|  | root tubers |
|  | swollen thorn |
|  | swollen thorns |
|  | trichilia |
|  | trichilium |
| **Extrafloral Nectar (EFN)** | efn |
|  | efns |
|  | extrafloral |
|  | foliar nectary |
|  | foliar nectaries |
| **Food Bodies** | Beltian body |
|  | Beltian bodies |
|  | food body |
|  | food bodies |
|  | Mullerian body |
|  | Mullerian bodies |
|  | pearl body |
|  | pearl bodies |
| **Seed Dispersal** | aril |
|  | elaiosome |
|  | elaiosomes |
|  | myrmecochory |
|  | myrmecochorous |
|  | myrmecochore |
|  | myrmecochores |
|  | seed dispersal |
|  | seed disperser |
