# Supplementary material for: Using text-mined trait data to test for cooperate-and-radiate co-evolution between ants and plants
Source: PLoS Comput Biol. 2019 Oct 3;15(10):e1007323. doi: 10.1371/journal.pcbi.1007323 (PMC6776258; doi:10.1371/journal.pcbi.1007323)
Supplement: S3 Table — (DOCX) [file pcbi.1007323.s008.docx]

*S3 Table.* For the BiSSE model, 95% credible intervals for speciation, extinction, and transition rates of lineages that do not (state 0) or do (state 1) associate mutualistically with plants.

|  | **lambda_0_** | **lambda_1_** | **mu_0_** | **mu_1_** | **q_01_** | **q_10_** |
| --- | --- | --- | --- | --- | --- | --- |
| **2.5%** | 0.094 | 0.38 | 0.093 | 0.0025 | 0.0061 | 0.33 |
| **97.5%** | 0.17 | 0.56 | 0.18 | 0.22 | 0.013 | 0.49 |
