# Supplementary material for: Using text-mined trait data to test for cooperate-and-radiate co-evolution between ants and plants
Source: PLoS Comput Biol. 2019 Oct 3;15(10):e1007323. doi: 10.1371/journal.pcbi.1007323 (PMC6776258; doi:10.1371/journal.pcbi.1007323)
Supplement: S4 Table — For the CID-4 model, not all transition rate categories are shown in the table because all 32 transition rates for this model are equal. The following transition rates are removed from all HiSSE models: q1B0A, q0B1A, q1A0B, and q0A1B, as they are dual transitions between both the observed trait and the hidden trait. (DOCX) [file pcbi.1007323.s009.docx]

*S4 Table.* Parameter estimate summary from HiSSE analyses for all models for the defense category (which includes trait information for domatia, EFN, and food bodies). For the CID-4 model, not all transition rate categories are shown in the table because all 32 transition rates for this model are equal. The following transition rates are removed from all HiSSE models: q1B0A, q0B1A, q1A0B, and q0A1B, as they are dual transitions between both the observed trait and the hidden trait.

|  | **Rate Class** | **lambda** | **mu** | **div** |  | **q10** | **q01** | **q0A1A** | **q0A0B** | **q1A0A** | **q1A1B** | **q0B0A** | **q0B1B** | **q1B1A** | **q1B0B** |
| --- | --- | --- | --- | --- | --- | --- | --- | --- | --- | --- | --- | --- | --- | --- | --- |
| **BiSSE no hidden states**  AIC 6735.356 | 0 | 0.496 | 0.457 | 0.0387 |  | 0.0413 | 0.00526 | not applicable | | | | | | | |
|  | 1 | 9.97E-02 | 0.103 | -3.44E-03 |  |  |  |  |  |  |  |  |  |  |  |
| **BiSSE null**  (lamba0=lamba1, mu0=mu1)  AIC 6724.252 | 0 | 0.443 | 0.405 | 0.0375 |  | 0.1795 | 0.0123 |  |  |  |  |  |  |  |  |
|  | 1 | 0.443 | 0.405 | 0.0375 |  |  |  |  |  |  |  |  |  |  |  |
| **CID-2 (Null-two) HiSSE** (lamba0A=lambda1A, mu0A=mu1A, lambda0B=lambda1B, mu0B=mu1B)  AIC 6634.781 | 0A | 0.915 | 0.874 | 0.0409 |  | not applicable | | 0.00329 | | | | | | | |
|  | 1A | 0.915 | 0.874 | 0.0409 |  |  |  |  |  |  |  |  |  |  |  |
|  | 0B | 0.0427 | 6.41E-09 | 0.0427 |  |  |  |  |  |  |  |  |  |  |  |
|  | 1B | 0.0427 | 6.41E-09 | 0.0427 |  |  |  |  |  |  |  |  |  |  |  |
| **CID-4 (Null-four) HiSSE** (lamba0A=lambda1A, mu0A=mu1A, lambda0B=lambda1B, mu0B=mu1B, lamba0C=lambda1C, mu0C=mu1C, lambda0D=lambda1D, mu0D=mu1D)  AIC 6560.723 | 0A | 0.0353 | 7.63E-11 | 0.0353 |  |  |  | 0.00596 (this model has 32 transitions, due to the added C and D rate classes) | | | | | | | |
|  | 1A | 0.0353 | 7.63E-11 | 0.0353 |  |  |  |  |  |  |  |  |  |  |  |
|  | 0B | 0.0315 | 7.55E-04 | 0.0308 |  |  |  |  |  |  |  |  |  |  |  |
|  | 1B | 0.0315 | 7.55E-04 | 0.0308 |  |  |  |  |  |  |  |  |  |  |  |
|  | 0C | 0.895 | 0.817 | 0.0776 |  |  |  |  |  |  |  |  |  |  |  |
|  | 1C | 0.895 | 0.817 | 0.0776 |  |  |  |  |  |  |  |  |  |  |  |
|  | 0D | 0.0644 | 0.0115 | 0.0529 |  |  |  |  |  |  |  |  |  |  |  |
|  | 1D | 0.0644 | 0.0115 | 0.0529 |  |  |  |  |  |  |  |  |  |  |  |
| **HiSSE 1 hidden state**  AIC 6660.837 | 0A | 0.589 | 0.572 | 0.0172 |  |  |  | 0.00775 | 0 | 0.114 | 0.00484 | 0 | 0 | 0.374 | 0 |
|  | 1A | 0.0641 | 0.150 | -0.0861 |  |  |  |  |  |  |  |  |  |  |  |
|  | 0B | 0 | 0 | 0 |  |  |  |  |  |  |  |  |  |  |  |
|  | 1B | 1.97 | 1.60 | 0.369 |  |  |  |  |  |  |  |  |  |  |  |
| **Full HiSSE**  AIC 6434.85 | 0A | 0.117 | 0.0948 | 0.0219 |  |  |  | 2.01E-04 | 3.53E-03 | 2.06E-09 | 2.06E-09 | 0.0609 | 0.0130 | 6.94E-03 | 0.107 |
|  | 1A | 0.0956 | 0.0795 | 0.0162 |  |  |  |  |  |  |  |  |  |  |  |
|  | 0B | 1.21 | 1.12 | 0.0899 |  |  |  |  |  |  |  |  |  |  |  |
|  | 1B | 0.105 | 0.0216 | 0.0832 |  |  |  |  |  |  |  |  |  |  |  |
