# Supplementary material for: Using text-mined trait data to test for cooperate-and-radiate co-evolution between ants and plants
Source: PLoS Comput Biol. 2019 Oct 3;15(10):e1007323. doi: 10.1371/journal.pcbi.1007323 (PMC6776258; doi:10.1371/journal.pcbi.1007323)
Supplement: S5 Table — For the CID-4 model, not all transition rate categories are shown in the table because all 32 transition rates for this model are equal. The following transition rates are removed from all HiSSE models: q1B0A, q0B1A, q1A0B, and q0A1B, as they are dual transitions between both the observed trait and the hidden trait. (DOCX) [file pcbi.1007323.s010.docx]

|  | **Rate Class** | **lambda** | **mu** | **div** |  | **q10** | **q01** | **q0A1A** | **q0A0B** | **q1A0A** | **q1A1B** | **q0B0A** | **q0B1B** | **q1B1A** | **q1B0B** |
| --- | --- | --- | --- | --- | --- | --- | --- | --- | --- | --- | --- | --- | --- | --- | --- |
| **BiSSE no hidden states**  AIC 7966.37 | 0 | 0.606 | 0.563 | 0.0422 |  | 0.0763 | 0.0164 | not applicable | | | | | | | |
|  | 1 | 0.019 | 0.0361 | -0.0171 |  |  |  |  |  |  |  |  |  |  |  |
| **BiSSE null**  (lamba0=lamba1, mu0=mu1)  AIC 7937.547 | 0 | 0.485 | 0.448 | 0.0368 |  | 0.1951 | 0.0285 |  |  |  |  |  |  |  |  |
|  | 1 | 0.485 | 0.448 | 0.0368 |  |  |  |  |  |  |  |  |  |  |  |
| **CID-2 (Null-two) HiSSE** (lamba0A=lambda1A, mu0A=mu1A, lambda0B=lambda1B, mu0B=mu1B)  AIC 7876.921 | 0A | 0.0450 | 1.05E-10 | 0.0450 |  | not applicable | | 0.00762 | | | | | | | |
|  | 1A | 0.0450 | 1.05E-10 | 0.0450 |  |  |  |  |  |  |  |  |  |  |  |
|  | 0B | 0.904 | 0.850 | 0.0538 |  |  |  |  |  |  |  |  |  |  |  |
|  | 1B | 0.904 | 0.850 | 0.0538 |  |  |  |  |  |  |  |  |  |  |  |
| **CID-4 (Null-four) HiSSE** (lamba0A=lambda1A, mu0A=mu1A, lambda0B=lambda1B, mu0B=mu1B, lamba0C=lambda1C, mu0C=mu1C, lambda0D=lambda1D, mu0D=mu1D)  AIC 7751.741 | 0A | 0.0431 | 1.81E-03 | 0.0412 |  |  |  | 0.0116 (this model has 32 transitions, due to the added C and D rate classes) | | | | | | | |
|  | 1A | 0.0431 | 1.81E-03 | 0.0412 |  |  |  |  |  |  |  |  |  |  |  |
|  | 0B | 0.0491 | 2.36E-10 | 0.0491 |  |  |  |  |  |  |  |  |  |  |  |
|  | 1B | 0.0491 | 2.36E-10 | 0.0491 |  |  |  |  |  |  |  |  |  |  |  |
|  | 0C | 0.590 | 0.526 | 0.0647 |  |  |  |  |  |  |  |  |  |  |  |
|  | 1C | 0.590 | 0.526 | 0.0647 |  |  |  |  |  |  |  |  |  |  |  |
|  | 0D | 1.56 | 0.526 | 1.03 |  |  |  |  |  |  |  |  |  |  |  |
|  | 1D | 1.56 | 0.526 | 1.03 |  |  |  |  |  |  |  |  |  |  |  |
| **HiSSE 1 hidden state**  AIC 7746.419 | 0A | 0.502 | 0.484 | 0.0180 |  |  |  | 0.0356 | 0 | 0.232 | 0.0681 | 0 | 0 | 0.220 | 0 |
|  | 1A | 0.0385 | 0.111 | -0.0728 |  |  |  |  |  |  |  |  |  |  |  |
|  | 0B | 0 | 0 | 0 |  |  |  |  |  |  |  |  |  |  |  |
|  | 1B | 6.85 | 6.70 | 0.148 |  |  |  |  |  |  |  |  |  |  |  |
| **Full HiSSE**  AIC 7568.277 | 0A | 0.0846 | 0.0531 | 0.0312 |  |  |  | 2.06E-09 | 0.00954 | 0.00236 | 0.00666 | 0.0705 | 0.0412 | 2.35E-02 | 0.0464 |
|  | 1A | 0.0161 | 3.33E-11 | 0.0161 |  |  |  |  |  |  |  |  |  |  |  |
|  | 0B | 1.095 | 0.966 | 0.130 |  |  |  |  |  |  |  |  |  |  |  |
|  | 1B | 0.298 | 0.303 | -0.00526 |  |  |  |  |  |  |  |  |  |  |  |

*S5 Table.* Parameter estimate summary from HiSSE analyses for all models for the seed dispersal category. For the CID-4 model, not all transition rate categories are shown in the table because all 32 transition rates for this model are equal. The following transition rates are removed from all HiSSE models: q1B0A, q0B1A, q1A0B, and q0A1B, as they are dual transitions between both the observed trait and the hidden trait.
